# Personality Factors and Depressive Configurations. An Exploratory Study in an Italian Clinical Sample

**DOI:** 10.3389/fpsyg.2017.00251

**Published:** 2017-03-03

**Authors:** Francesca Straccamore, Simona Ruggi, Vittorio Lingiardi, Raffaella Zanardi, Sara Vecchi, Osmano Oasi

**Affiliations:** ^1^Department of Dynamic and Clinical Psychology, Sapienza University of RomeRome, Italy; ^2^Department of Psychology, Università Cattolica del Sacro CuoreMilan, Italy; ^3^Department of Clinical Neurosciences, Vita-Salute San Raffaele University (UniSR)Milan, Italy

**Keywords:** dependency, self-criticism, vulnerability, depressive personality, depressive symptomatology

## Abstract

**Introduction:** This study focuses on the relationship between personality configurations and depressive experiences. More specifically, the aim of this study is to investigate the relationship between self-criticism and dependency and personality styles or disorders, exploring the association between personality features and depressive symptoms. The two-configurations model of personality developed by Blatt (2004, 2008) is adopted as a reference point in sharing a valid framework and in understanding the results.

**Methods:** Five instruments are administered to 51 participants with a diagnosis of depressive disorder, in accordance with DSM-IV-TR (American Psychiatric Association, 2000): Self-criticism and dependency dimensions of depression are measured with the Depressive Experiences Questionnaire (DEQ); self-reported depression is assessed with the Beck Depression Inventory-II (BDI-II); observer-rated depression is assessed with the Hamilton Depression Rating Scale (HDRS); personality is assessed with the Clinical Diagnostic Interview (CDI) and the Shedler Westen Assessment Procedure-200 (SWAP-200).

**Results:** Only self-criticism, and not dependency, is associated with depressive symptoms. In addition, the SWAP Borderline PD Scale and the Dysphoric: Emotionally dysregulated Q-factor emerge as significant in predicting depression.

**Conclusions:** Findings support the assumption that depressive personality configurations can enhance the vulnerability to developing depression. Theoretical and clinical implications of these results are discussed.

## Introduction

Over the last few decades, from a psychoanalytic and cognitive developmental perspective, Blatt (Blatt, 1974, 1990, 2004, 2008; Blatt and Shichman, 1983; Blatt and Maroudas, 1992) developed a two-polarities theoretically and empirically supported model of personality that focuses on interpersonal relatedness and self-definition as central coordinates in personality development and psychopathology (Luyten and Blatt, 2013). In normal personality development, these two developmental lines evolve throughout life in an interactive, reciprocally balanced or dialectic transaction. Conversely, psychopathology can be conceptualized as involving an overemphasis and exaggeration of one of these developmental lines and the defensive avoidance of the other (Blatt et al., 2001). Exaggerated emphasis on one of these two normal developmental lines may lead to an extreme personality trait: Dependency or self-criticism. Therefore, these two developmental dimensions of relatedness and self-definition provide a theoretical matrix that identifies continuities between variations of normal personality organization and different forms of psychopathology (Blatt, 2008).

Luyten and Blatt (2013) conceptualized maladaptive expressions of dependency and self-criticism as *transdiagnostic vulnerability factors*, which may also partly explain the high comorbidity among “symptom” and “personality” disorders; high levels of these personality dimensions confer vulnerability to distress in both clinical and community samples. A growing body of empirical and clinical research attests to the relevance of self-criticism and dependency as forms of personality vulnerability in the context of depression and with regards to its treatment (Choi et al., 2015); according to the specific “vulnerability hypothesis” (Zuroff et al., 2004), these personality characteristics represent predispositions that may confer vulnerability to anaclitic (dependent) or introjective (or self-critical) depression, respectively. Blatt used “anaclitic” and “introjective” both to denote types of depressive *states* and to refer to the types of *personalities* that are especially vulnerable to such states (Zuroff and Mongrain, 1987).

Anaclitic depression affects individuals that are primarily responsive to the disruptions of gratifying interpersonal relationships (e.g., object loss). This configuration of depression is characterized by feelings of loneliness, weakness, helplessness, intense fear of being unloved, unwanted, uncared for, and abandoned. The various symptoms express a desperate neediness; somatic complaints often express the wish to be soothed and comforted. Introjective depression affects individuals that are primarily responsive to the disruptions of an effective and essentially positive sense of self (e.g., failure). It is characterized by self-criticism, feelings of unworthiness, inferiority, failure, and guilt. These individuals can be critical, perfectionist, highly competitive, and hard-working; they have a chronic fear of being disapproved of something and criticized, as well as of losing the approval and acceptance of significant others (Blatt and Shichman, 1983; Blatt and Zuroff, 1992; Blatt, 2004).

This theoretical model provides a way of conceptualizing psychopathology in the first place and depression in the second that has important implications for understanding the etiology of disorders as well as for therapeutic intervention. As stated by Luyten and Blatt (2007), a dimensional approach, with depression situated on a continuum ranging from mild dysphoria to full-blown clinical depression, seems to be more valid than the DSM “count/cutoff” method (Westen et al., 2006).

Right from his initial studies, Blatt encouraged clinicians to investigate cognitive styles, affective aspects, interpersonal functioning and personality configurations related to the anaclitic and the introjective dimensions of depression (Blatt et al., 1976). As stated by Blatt et al. (1982, p. 121), “Although these dimensions of depression were originally identified in non-clinical subjects, they appear to provide a typology for understanding aspects of depression in psychiatric patients.”

Research findings have indicated that the *Depressive Experiences Questionnaire*(DEQ; Blatt et al., 1976) can be used to assess these two fundamental personality dimensions, in particular to measure adaptive as well as maladaptive aspects of the two fundamental developmental processes—that is to say, of both interpersonal relations and self-definition (Blatt et al., 1995; Rude and Burnham, 1995; Blatt, 2004, 2008; McBride et al., 2006).

A considerable amount of research has examined the relationship between anaclitic and introjective personality organizations and depressive disorders (Blatt et al., 1982; Zuroff and Mongrain, 1987; Besser and Priel, 2005; Mongrain and Leather, 2006; Luyten et al., 2007; Campos et al., 2011; Miller and Hilsenroth, 2016). With regard to the personality configurations, to the best of our knowledge, an extensive corpus of research has studied the relationship between these two types of personality organization and the Five-Factor model of personality (Zuroff, 1994; Dunkley et al., 2006; Henriques-Calado et al., 2013). However, only a few studies have investigated the relationship between the two types of depressive experience and specific personality styles or disorders.

Blatt and Shichman (1983) predicted that psychopathology in the anaclitic configuration ranges from an infantile syndrome to hysteria, whereas psychopathology in the introjective configuration includes paranoia, obsessive-compulsive syndrome, introjective depression, and phallic narcissism. Pilkonis (1988) studied the personality prototypes among depressed patients and found that excessive autonomy was related to obsessive-compulsive features, defensive separation, and lack of interpersonal sensitivity, whereas excessive dependency was associated with “anxious attachment” and features typically connected with borderline personality disorders. Ouimette et al. (1994) examined the relationship between Beck's constructs of sociotropy/autonomy and Blatt's constructs of dependency and self-criticism and DSM-III-R personality disorders. They found that dependency was significantly correlated with dependent, histrionic, and borderline traits, whereas self-criticism was significantly associated with paranoid, narcissistic, obsessive-compulsive, schizoid, and passive-aggressive traits. Moreover, findings of this study suggested that the autonomous/self-critical style may encompass a broader, less specific, range of psychopathology. Morse et al. (2002) studied the relation of sociotropy and autonomy to DSM-III-R personality disorders. They found that histrionic and dependent personality disorders were related to sociotropy, and paranoid, schizoid, schizotypal, and passive-aggressive PD traits were related to autonomy. Borderline, narcissistic, avoidant, and self-defeating PD traits were related significantly to both sociotropy and autonomy. More recently, Ryder et al. (2008) examined the relation between DSM-IV PDs and two personality styles: The achievement style and the affiliation style. Findings suggested that the affiliation style was related to histrionic, dependent, and depressive PDs, whereas the achievement style was related to paranoid, schizoid, narcissistic, antisocial, obsessive-compulsive, negativistic, and depressive PDs, but also with schizotypal, borderline, and avoidant PDs.

Generally speaking, with regard to gender differences and their cultural and psychological implications, the findings in literature (Campos et al., 2013) suggested that dependency is more represented in female samples, while self-criticism is more represented in male samples. Blatt (2008) explained this gendered pattern with the fact that women's and men's experience in personality development are different. In particular, women are argued to place more emphasis on issues related to interpersonal relatedness, especially in terms of giving and receiving care, affection, and love. On the contrary, men tend to place more emphasis on self-definition, especially in terms of individualistic self-assertion.

The general aim of the current study is to investigate the relationship between personality factors and depressive configurations. In particular, we expect to find that in our Italian clinical sample some specific personality factors will likewise prove correlated with the two different depressive configurations described by Blatt. When possible, we suggest how personality factors are predictive of depressive conditions.

## Materials and methods

### Participants

A total of 51 subjects were initially recruited for the study, 17 (33.3%) men and 34 (66.7%) women (mean age total = 51.59 years, male = 51.76 years, female = 51.50 years; SD total = 11.68, male = 13.96, female = 10.59); all of which undergoing an episode of depressive disorder at that time, according to criteria of the Diagnostic and Statistical Manual of Mental Disorders (DSM-IV-TR; American Psychiatric Association, 2000); in particular, 46 (90.2%) of the patients were diagnosed with major depressive disorder, 4 (7.8%) with dysthymic disorder and 1 (2%) with double depression. All DSM-IV initial diagnoses were formulated by clinicians with over two decades of experience. At the time of assessment all participants were in the midst of an unipolar depressive episode and were capable of and willing to give informed consent for assessment. Exclusion criteria included: Major depression with psychotic features and specific additional psychiatric disorders such as a history of schizophrenia, bipolar I or II disorders; past hypomanic or manic episodes; severe alcohol or drug addiction; pregnancy; organic brain syndrome; mental retardation or other cognitive impairment that might interfere with the accuracy of the assessment or competency to give informed consent; presence of specific physical illnesses; presence of a clinical state inconsistent with participating in the research protocol (e.g., current active suicide potential or need for immediate treatment). 11 patients were excluded based on these criteria. Of the 51 participants who took part in the study, 40 were out-patients recruited from mental health centers in Rome and the surrounding area, 11 were in-patients recruited from the mood disorders unit of the St. Raffaele Hospital in Milan. 35 participants (68.6%) had had (at least) three episodes, whereas 9 (17.6%) had had (at least) two and finally 7 (13.7%) had experienced only one prior episode. 6 participants (11.76%) were diagnosed with a comorbid anxiety disorder. 47 of the 51 patients (92.2%) were receiving psychotropic medication. In socioeconomic terms, most of the patients came from middle class families or higher, were well-educated and had an average intelligence or higher. Participants were predominantly of Italian nationality. Descriptive data for the participants is presented in Table 1.

**Table 1:** **Descriptive data for the participants**.

**Demographic factors**	***N***	**%**
**MARITAL STATUS**		
Single	15	29.4
Married	19	37.3
Divorced	15	29.4
Widowed	2	3.9
**EMPLOYED EDUCATION**	14	27.45
Elementary	16	31.4
Some form of Higher	23	45.1
Degree	12	23.5
**SOCIO-ECONOMIC STATUS**		
Low	15	29.4
Medium	30	58.8
High	6	11.8
**NATIONALITY**		
Italian	50	99.98

*N = 51*.

### Materials

#### Beck Depression Inventory—second edition (BDI-II)

The BDI-II is a 21-item measurement of the cognitive, affective, motivational, and somatic symptoms of depression. Each item has a four-point (0–3), likert style rating scale. The BDI-II is scored by summing the ratings for the 21 items, with the maximum score being 63. BDI-II scores ranging from 0 to 13 indicate *normal—minimal* depressive levels; scores ranging from 14 to 18 indicate *mild—moderate* depressive levels; scores ranging from 19 to 29 indicate *moderate—severe* depressive levels; scores ranging from 30 to 63 indicate *extremely severe* depressive levels. Scores below 12 are not considered clinically significant. Studies into the factorial structure of the BDI-II usually discern two subscales: The *Somatic-Affective factor* contains 12 items that reflect the affective, somatic, and vegetative symptoms of depression; the *Cognitive factor* contains 9 items that reflect cognitive symptoms of depression (Beck et al., 1996; Steer et al., 1999). Cronbach alphas for the current study attest to excellent levels of internal consistency (ICC) and reliability for the BDI-II total score (α = 0.918), and good ICC for the BDI-COGN (α = 0.872), and the BDI-SOMA (α = 0.847).

#### Hamilton depression rating scale (HAMD)

The HAMD consists of 21 items that largely assess somatic and neurovegetative aspects of depression (Hamilton, 1960). Each item is measured on five-point or three-point scales and no difference is made between intensity and frequency of symptoms. As recommended by Hamilton (1960), the total HDRS score was obtained by summing the ratings for the first 17 items only. Although there has been a relatively problematic lack of standardization in administration instructions and scoring criteria (Reynolds and Kobak, 1995; Williams, 2001), the following scoring conventions have emerged over time: Scores below 6 are generally considered to indicate normal functioning, scores ranging from 7 to 17 indicate *mild* depressive levels; scores ranging from 18 to 24 indicate *moderate* depressive levels; finally, scores ranging 25 or greater indicate *severe* depressive levels (Endicott et al., 1981). In this study only the general factor described by Hamilton (1967) was considered as reflecting the severity of depressive symptoms^1^; the ICC for the HAMD was high (α = 0.798).

#### Depressive Experiences Questionnaire (DEQ)

The DEQ (Blatt et al., 1976) is a 66 item questionnaire in which individuals rate themselves on a wide range of life experiences frequently associated with depression but not considered to be directly symptomatic expressions of depression (Blatt et al., 1976; Blatt, 2004). Rather than depressed mood or characteristics of other states, the DEQ assesses primarily stable, continuous personality characteristics that could be interpreted as measures of vulnerability to experiencing of two certain types of mood (Zuroff et al., 1990). Participants are asked to rate each item on a seven-point Likert-type scale ranging from *strongly disagree* (1) to *strongly agree* (7). Three major factors emerged from the factor analysis:

#### Dependency (factor 1)

The dependency factor (or *Interpersonal Concerns* factor) (Blatt, 2004) contains items that are primarily externally directed, involves interpersonal relations, and reflects concerns with abandonment and separation, the feeling of being unloved, and wanting to be close to, related to, and dependent upon others, helplessness, fear of loss, and difficulty in dealing with anger.

#### Self-criticism (factor 2)

The self-criticism factor contains items that are primarily internally directed and indicate concerns about failure, guilt, self-blame, emptiness, hopelessness, dissatisfaction, insecurity, failure to meet expectations and standards, ambivalence about self and others, and distorted or depreciated sense of self and other.

#### Efficacy (factor 3)

The Efficacy factor consists of items that reflect a sense of confidence about one's resources and capacities and of personal effectiveness and competence, high standards and personal goals, a sense of responsibility, inner strength, feelings of independence, and a sense of pride and satisfaction in one's accomplishments.

Blatt et al. (1982, p. 122) suggested that in a clinical context *Efficacy* “seems to reflect a hypomanic denial of difficulties, particularly those deriving from issues of dependency.” Since the majority of studies involving the DEQ don't take this factor into account (Zuroff and de Lorimier, 1989; Ouimette et al., 1994; Desmet et al., 2006; Luyten et al., 2007; see Campos et al., 2011), in the present study it has likewise been omitted from all analyses.

Subsequent researches (Blatt et al., 1995; Rude and Burnham, 1995; Blatt, 2004) identified two subscales, *dependence* (or *neediness*) and *relatedness* (or *connectedness*)—within the dependency factor—respectively associated with less and more mature (or less dysfunctional) levels of interpersonal concern.

In the present study, as recommended by Blatt et al. (1995), the standard scoring system of the DEQ and the factor weight coefficients were used; subjects' factor scores are *z* scores. The ICC in the current study was good for the DEQ-SC (α = 0.755) and the DEQ-EFF (α = 0.768), acceptable for the DEQ-DEP (α = 0.626), poor for the *neediness subscale* (α = 0.524), and unacceptable for the *dependency subscale* (α = 0.407). For this reason, in the present investigation, the *neediness* and the *dependency* subscales were omitted from all analyses.

#### Clinical Diagnostic Interview (CDI)

In contrast to a structured interview, the CDI (Shedler et al., 2014), is what might be called a *systematic clinical interview*, that should be conducted as a clinical interview. It is a narrative-based interview because it elicits narratives about patients' symptoms and life histories and focuses on specific examples of emotionally salient experiences (Westen and Weinberger, 2004; Westen and Muderrisoglu, 2006). Although the CDI includes a number of direct questions (e.g., about characteristic moods), it does not primarily ask patients to describe their personalities. Rather, it asks them to tell narratives about themselves, their lives and their problems that allow the interviewer to make judgments about their characteristic ways of thinking, feeling, regulating emotions and impulses, experiencing themselves and others, and so forth. The interview begins, as in any standard clinical interview, by asking patients what brought them to treatment, with the interviewer probing for details about severity, frequency, duration, and history of symptoms and concerns. The interviewer then asks patients about a wide range of significant relationships and experiences from the past and present (e.g., parents, siblings, romantic relationships, friendships, school, and work experiences), about particularly stressful or difficult times in their recent lives (to obtain information about how the patients appraises and copes with difficult circumstances), about their moods and emotions, and about their characteristic ways of thinking (to obtain data on subclinical thinking disturbances). For each of these categories of relationships or experiences, the interviewer asks the patient to describe two to three specific incidents.

#### Shedler Westen Assessment Procedure-200 (SWAP-200)

The SWAP-200 is a personality assessment instrument that consists of 200 descriptive statements of personality yielding a range of meaningful information about cognitive, emotional, motivational, and relational functioning (Westen and Shedler, 1999a,b; Shedler et al., 2014). These items are close to the observed data and require minimal inference about internal processes. The SWAP is based on the Q-sort method: The rater is required to sort the statements into eight categories based on the degree to which they describe the patient, from 7 (*highly descriptive*) to 0 (*not descriptive* or *non-applicable to the patient* or *irrelevant to describing this patient's personality*). In order to maximize reliability and minimize error variance, the distribution of scores is fixed: Only eight items can be given the highest score of 7, 10 are scored at 6, 12 are scored at 5, and so forth (Westen and Shedler, 2007). The SWAP provides a common vocabulary that organizes clinical observations and inferences about a patient's personality and psychological functioning (Lingiardi et al., 2006; Gazzillo et al., 2013). Following the transformation into standardized *T* scores, 22 scales are obtained: 11 scales of personality disorders consistent with the description on DSM-IV-TR Axis II diagnoses (the Personality Disorder or *PD scores*) and 11 scales for personality factors empirically derived from descriptions of the patient according to their therapists through Q-factor analysis (*Q factors*). When the *T* score for a given prototype is 60 or more, the patient is considered to have that personality disorder, whereas if the score is 55 to 59, the patient is considered to have features of the disorder but not a full-blown disorder. The psychometric properties of reliability and validity of the SWAP have been repeatedly demonstrated in several studies (Shedler and Westen, 2007).

### Procedures

This research project was approved by the Ethics Committee of the Azienda U.S.L. Rome E with the following signature: Number CE/10/2014. All participants signed a written informed consent form and received an informative sheet on the research. None were paid or compensated for their participation. All of them were assessed by the same Ph.D. level research-clinician. Data was collected in approximately four individual sessions, and instructions were presented in written form. After having gathered their informed consent, patients were asked to complete the BDI-II and the DEQ. Then, the CDI was administered in approximately three or four 45–50 min sessions, including feedback to the patient at the end of the last interview. Each interview was audiotaped and transcribed. After completing the CDI, the interviewer sorted the 200 items of the SWAP-200 according to the degree to which they applied to the patient. Clinicians who administered the HAMD were blind to BDI-II scores. Data was collected over a period of 2 years (from January 2014 to 2016).

### Data analyses

Firstly, means and standard deviations for DEQ factors and depressive measures were calculated. Secondly, the presence of personality disorders was assessed. Thirdly, correlations (Pearson's *r*) between the DEQ scales and all study measures were computed. Fourthly, in order to examine the unique associations between the personality predispositions and the depressive symptoms, a series of regression analyses was performed. Finally, gender differences in depressive symptomatology and personality variables were investigated. All statistical analyses were performed using IBM SPSS v20.0 software.

## Results

### Descriptive analyses

The scores of BDI-II, HAMD and DEQ didn't reveal a reasonably normal distribution; data was considered within the limits of a normal distribution if skewness and kurtosis did not exceed 1 (in absolute value) (Peat and Barton, 2005).

Means and standard deviations for the DEQ scales, the BDI-II and its subscales along with the HAMD are presented in Table 2.

**Table 2:** **Means and standard deviations for study measures**.

**Measure**	**M**	***SD***
**DEQ**		
DEQ-DEP	–0.12	0.96
DEQ-SC	0.34	1.09
**BDI-II**	25.37	12.8
BDI-II SOMA	14.75	7.2
BDI-II COGN	10.65	6.35
**HAMD**	19.41	7.23

*N = 51. DEQ-DEP, Dependency scale of the DEQ; DEQ-SC, Self- criticism scale of the DEQ. BDI SOMA, Somatic dimension of the BDI; BDI COGN, Cognitive dimension of the BDI*.

Since in the existing literature there is a lack of normative information based on standardized scores of large clinical samples for the interpretation of the DEQ, one of the most commonly used methods is comparing the clinical sample with normative samples. In a large sample the means for *dependency* and *self-criticism* were −0.10 and −0.19, and the *SD*s were 0.83 and 0.87 (Zuroff et al., 1990). Comparing the sample means suggests that participants in this study were somewhat biased toward lower levels of *dependency* and higher levels of *self-criticism*. With regard to the severity of depressive symptoms, for these participants the mean BDI-II total score was in the moderate—severe range. Similarly, the mean HAMD score was in the moderate range.

The presence of personality styles or disorders was assessed using the SWAP-200. With regard to *PD* factors, of the 51 patients included in this study, 19 met criteria for one or more personality disorders, 22 had significant features of at least one personality disorder, and 10 had minor or no features of any personality disorder. More specifically, of those meeting criteria for personality disorders, 24 subjects presented Cluster C traits, 12 presented Cluster B traits, and 5 presented Cluster A traits.

With regard to *Q* factors, 26 subjects met criteria for one or more Q factor diagnosis and 25 subjects had significant features of one or more Q factors. In particular, the most prevalent Q factors were: Dysphoric: High-functioning neurotic (13 subjects); Dysphoric: Emotionally dysregulated (11 subjects); Narcissistic (9 subjects); Avoidant (6 subjects).

### Correlation analyses

#### DEQ scales and depressive measures

The Pearson correlations between the DEQ scales and the measures of depression obtained are reported in Table 3.

**Table 3:** **Pearson correlations between DEQ scales and measures of depression**.

**Scale**	**DEQ-DEP**	**DEQ-SC**	**HAMD**	**BDI-II**	**BDI-SOMA**
2. DEQ-SC	0.239				
3. HAMD	0.116	0.312^*^			
4. BDI-II	0.012	0.444^**^	0.576^**^		
5. BDI-SOMA	0.005	0.386^**^	0.519^**^	0.951^**^	
6. BDI-COGN	0.018	0.460^**^	0.576^**^	0.938^**^	0.785^**^

*N = 51. All variables were standardized before analyses*.

* *p < 0.05 (two tailed tests)*.

** *p < 0.01 (two tailed tests)*.

Firstly, it can be seen that for these participants, probably due to the use of the standard DEQ (Zuroff et al., 2004), the correlation between dependency and self-criticism was not significant. Secondly, it seems important that self-criticism showed a higher positive correlation than dependency with BDI-II—in particular with its cognitive scale, but also with its somatic scale—and with HAMD scores. Thirdly, the HAMD showed a positive correlation with the BDI-II and its subscales.

#### DEQ scales and SWAP factors

With respect to PD factors, dependency was positively correlated both with Schizoid (*r* = 0.279, *p* < 0.05), Avoidant (*r* = 0.427, *p* < 0.01), and Dependent (*r* = 0.509, *p* < 0.01) PD factors; conversely, it showed a negative correlation both with Narcissistic (*r* = −0.370, *p* < 0.01) and Antisocial (*r* = −0.345, *p* < 0.01) PD factors. Self-criticism was positively correlated with Avoidant (*r* = 0.415, *p* < 0.01) and Dependent (*r* = 0.311, *p* < 0.01) PD factors too (although self-criticism was less correlated than dependency) (Table 4). With respect to Q factors, dependency had a positive correlation with Dysphoric^2^ (*r* = 0.436, *p* < 0.01), Avoidant (*r* = 0.361, *p* < 0.01), and Dependent (*r* = 0.403, *p* < 0.01) Q factors. Self-criticism showed a positive correlation with Dysphoric (*r* = 0.495, *p* < 0.01), Avoidant (*r* = 0.317, *p* < 0.01) and Dysphoric: Emotionally dysregulated (*r* = 0.347, *p* < 0.01) Q factors (Table 4).

**Table 4:** **Pearson correlations between DEQ scales and SWAP-200 factors**.

**Scale**	**DEQ-DEP**	**DEQ-SC**
***SWAP-200 PD FACTORS***		
Schizoid	0.279^*^	0.221
Narcissistic	–0.370^**^	–0.073
Antisocial	–0.345^*^	–0.098
Avoidant	0.427^**^	0.415^**^
Dependent	0.509^**^	0.311^*^
***SWAP-200 Q FACTORS***		
Dysphoric	0.436^**^	0.495^**^
Dysphoric: avoidant	0.361^**^	0.317^**^
Dysphoric: emotionally dysregulated	0.040	0.347^*^
Dysphoric: dependent	0.403^**^	0.216

*N = 51. All variables were standardized before analyses*.

* *p < 0.05 (two tailed tests)*.

** *p < 0.01 (two tailed tests)*.

#### SWAP factors and depressive measures

To explore the association between SWAP factors and depressive measures further correlation analyses were performed. Among PD factors, the following significant correlations were obtained.

Schizotypal PD factor with BDI-Cogn (*r* = 0.324, *p* < 0.05), Borderline PD factor with BDI-II (*r* = 0.357, *p* < 0.05), BDI-Cogn (*r* = 0.444, *p* < 0.01); Avoidant PD factor with BDI-II (*r* = 0.355, *p* < 0.05), BDI-Cogn (*r* = 0.380, *p* < 0.01), BDI-Soma (*r* = 0.293, *p* < 0.05); Dependent PD factor with BDI-II (*r* = 0.339, *p* < 0.05), BDI-Cogn (*r* = 0.368, *p* < 0.01), BDI-Soma (*r* = 0.276, *p* < 0.05). No PD factor showed a significant correlation with HDRS (Table 5). Among Q factors, the subsequent significant associations were found: Dysphoric with BDI-II (*r* = 0.527, *p* < 0.01), BDI-Soma (*r* = 0.432, *p* < 0.01), BDI-Cogn (*r* = 0.571, *p* < 0.01), HAMD (*r* = 0.339, *p* < 0.01); Obsessional Q factor with BDI-II (*r* = −0.411, *p* < 0.01); BDI-Cogn (*r* = −0.503, *p* < 0.01), BDI-Soma (*r* = −0.283, p < 0.05), HAMD (*r* = −0.335, *p* < 0.05); Avoidant Q factor with BDI-II (*r* = 0.286, *p* < 0.05), BDI-Cogn (*r* = 0.298, *p* < 0.05); Dysphoric: Emotionally dysregulated Q factor with BDI-II (*r* = 0.532, *p* < 0.01), BDI-Cogn (*r* = 0.630, *p* < 0.01), BDI-Soma (*r* = 0.386, *p* < 0.01), HAMD (*r* = 0.471, *p* < 0.01); Dependent Q factor with BDI-Cogn (*r* = 0.279, *p* < 0.05); High Functionig Depressive Personality Q factor with BDI-II (*r* = −0.351, *p* < 0.05), BDI-Cogn (*r* = −0.436, *p* < 0.01), HAMD (*r* = −0.335, *p* < 0.05); (Table 5).

**Table 5:** **Pearson correlations between SWAP factors and measures of depression**.

**Factors**	**BDI-II**	**BDI-SOMA**	**BDI-COGN**	**HAMD**
***SWAP-200 PD FACTORS***				
Schizotypal	0.261	0.172	0.324^*^	0.233
Borderline	0.357^*^	0.239	0.444^**^	0.234
Avoidant	0.355^*^	0.293^*^	0.380^**^	0.213
Dependent	0.339^*^	0.276^*^	0.368^**^	0.148
***SWAP-200 Q FACTORS***				
Dysphoric	0.527^**^	0.432^**^	0.571^**^	0.339^*^
Obsessional	–0.411^**^	–0.283^*^	–0.503^**^	–0.335^*^
Dysph.: avoidant	0.286^*^	0.244	0.298^*^	0.152
Dysph.: em.dysr.	0.532^**^	0.386^**^	0.630^**^	0.471^**^
Dysph.: depend.	0.242	0.181	0.279^*^	–0.045
High Fun.Dep.P.	–0.351^*^	–0.236	–0.436^**^	–0.335^*^

*N = 51. All variables were standardized before analyses*.

* *p < 0.05 (two tailed tests)*.

** *p < 0.01 (two tailed tests)*.

### Regression analyses

#### Relationship between depressive measures

To test the hypothesis that personality dimensions play an important role in predicting depression, a series of regression analyses were performed in order to determine the potential effect of personality variables on severity of depression. A chi-square test between the HAMD and the BDI-II showed that, differently from some findings in literature (Duberstein and Heisel, 2007), in our study the way in which depressive symptomatology is self-reported and clinician-rated is very similar [$X_{\left(1\right)}^{2}$ = 16.579, *p* = 0.000]. Since all predictors showed a higher correlation with the BDI-II than the HAMD, BDI-II was chosen as the dependent variable. As predictors, only the variables significantly correlated with the dependent variable, the BDI-II, were considered in all regression analyses.

Primarily, in order to determine the unique proportion of variance explained by each set of predictors, regression analyses were conducted for every set of criterion variables separately, that is to say for DEQ personality variables, SWAP-200 *PD* factors, and SWAP-200 *Q* factors, respectively.

#### Relationship between DEQ scales and severity of depression

Firstly, the relationship between dependency and self-criticism and severity of depression was examined, entering each DEQ scale as the predictor variable in the regression analyses.

As can be seen in Table 6, only self-criticism—and not dependency—showed significant associations with all measures of depressive symptomatology.

**Table 6:** **Regression analyses of severity of depression: DEQ-SC and DEQ-DEP**.

**Predictor variable**	**BDI-II**			**BDI-SOMA**			**BDI-COGN**			**HAMD**		
	**β**	***t***	***p***	**β**	***t***	***p***	**β**	***t***	***p***	**β**	***t***	***p***
DEQ-SC	**0.444**	**3.470**	**0.001**	**0.386**	**2.931**	**0.005**	**0.460**	**3.623**	**0.001**	0.312	2.297	0.026
DEQ-DEP	0.012	0.086	0.932	0.005	0.032	0.974	0.018	0.125	0.901	00.116	0.818	0.417

*N = 51. Bold character indicates the significant values*.

Next, as in Desmet et al. (2006), the associations between the DEQ personality factors and separate BDI-II symptoms were explored (Table 7).

**Table 7:** **Regression analyses of BDI-II symptoms: DEQ-SC and DEQ-DEP**.

**BDI-II symptoms**	**DEQ-SC**			**DEQ-DEP**		
	**β**	***t***	***p***	**β**	***t***	***p***
Sadness^b^	0.105	0.737	0.465	−0.149	−1.056	0.296
Pessimism^b^	**0.379**	**2.869**	**0.006**	0.058	0.407	0.686
Past failure^b^	**0.436**	**3.391**	**0.001**	0.043	0.301	0.765
Loss of pleasure^a^	0.073	0.511	0.612	−0.102	−0.718	0.476
Guilty feelings^b^	**0.406**	**3.112**	**0.003**	**0.383**	**2.898**	**0.006**
Punishment feelings^b^	**0.341**	**2.540**	**0.014**	−0.085	−0.596	0.554
Self-dislike^b^	**0.392**	**2.987**	**0.004**	0.083	0.585	0.561
Self-criticalness^b^	**0.437**	**3.400**	**0.001**	0.008	0.058	0.954
Suicidal thoughts^b^	0.219	1.572	0.122	−0.075	−0.529	0.599
Crying	0.220	1.577	0.121	0.093	0.652	0.517
Agitation^a^	−0.039	−0.277	0.783	−0.131	−0.929	0.358
Loss of interest^a^	**0.328**	**2.430**	**0.019**	−0.008	−0.054	0.957
Indecisiveness^b^	**0.306**	**2.248**	**0.029**	0.044	0.307	0.760
Worthlessness^b^	0.204	1.460	0.151	−0.081	−0.566	0.574
Loss of energy^a^	0.260	1.882	0.066	−0.021	−0.148	0.883
Changes in sleeping^a^	0.208	1.487	0.143	0.171	1.214	0.230
Irritability^a^	0.237	1.706	0.094	0.026	0.180	0.858
Changes in appetite^a^	**0.363**	**2.723**	**0.009**	−0.036	−0.249	0.804
Concentration difficulty^a^	**0.299**	**2.194**	**0.033**	−0.087	−0.608	0.547
Tiredness or fatigue^a^	**0.321**	**2.375**	**0.021**	−0.107	−0.750	0.457
Loss of interest in sex^a^	0.221	1.586	0.119	0.134	0.947	0.348

*N = 51*.

a *Symptom belonging to the somatic symptom cluster*.

b *Symptom belonging to the cognitive symptom cluster*.

*Bold character indicates the significant values*.

As can be seen, self-criticism showed significant positive associations with “Pessimism,” “Past Failure,” “Guilty Feelings,” “Punishment Feelings,” “Self-Dislike,” “Self-Criticalness,” “Loss of Interest,” “Indecisiveness,” “Change in Appetite,” “Concentration Difficulty,” “Tiredness or Fatigue.” Conversely, dependency showed significant positive associations only with “Guilty Feelings.”

Furthermore, the same type of regression analysis was performed to explore associations between the DEQ personality factors and separate HAMD symptoms. While self-criticism showed significant positive associations with “Depressed Mood” (β = 0.396, *t* = 3.022, *p* = 0.004), “Anxiety Psychic” (β = 0.294, *t* = 2.157, *p* = 0.036) and “Anxiety Somatic” (β = 0.336, *t* = 2.496, *p* = 0.016), dependency showed significant positive associations with “Retardation” (β = 0.298, *t* = 2.188, *p* = 0.033), “Anxiety Psychic” (β = 0.321, *t* = 2.373, *p* = 0.022), “Depersonalization and Derealization” (β = −0.358, *t* = −2.685, *p* = 0.010).

#### Relationship between SWAP-200 factors and severity of depression

To explore the association between SWAP-200 factors and severity of depression further regression analyses were performed. Initially, all predictor variables were entered separately, each one in a single block. The following PD factors showed significant associations with the BDI-II: Borderline (β = 0.357, *t* = 2.679, *p* = 0.010), Avoidant (β = 0.355, *t* = 2.662, *p* = 0.010), Dependent (β = 0.339, *t* = 2.523, *p* = 0.015). Among the Q factors, the variables that showed significant associations with the BDI-II were: Dysphoric: Emotionally dysregulated (β = 0.532, *t* = 4.403, *p* = 0.000), Obsessive (β = −0.411, *t* = −3.154, *p* = 0.003), High Functioning Depressive Personality (β = −0.351, *t* = −2.628, *p* = 0.011) Q factors, Avoidant (β = 0.286, *t* = 2.089, *p* = 0.042).

The predictor variables, previously considered separately, were in a second step entered simultaneously, in standard regression analyses. Among PD factors, only the Borderline PD factor (β = 0.299, *t* = 2.144, *p* = 0.037) showed significant associations with the BDI-II. Among Q factors, only the Dysphoric: Emotionally dysregulated (β = 0.680, *t* = 2.676, *p* = 0.010) showed a significant association with the BDI-II.

#### Relationship between all personality variables and severity of depression

All predictor variables, previously considered separately—DEQ factors and SWAP PD and Q factors—were entered simultaneously, first in a standard regression analysis, then in a stepwise regression analysis. While in the standard regression analyses no factor resulted associated with BDI-II, in the stepwise regression both Dysphoric: Emotionally dysregulated Q factor (β = 1.016, *t* = 4.006, *p* = 0.000) and Borderline PD factor (β = −0.545, *t* = −2.148, *p* = 0.037) showed an association with BDI-II.

### Gender differences in personality factors and depressive measures

To explore the presence of gender differences associated with personality variables and depressive measures, a series of comparisons between groups was computed. Given that, in the current study, consistent with the literature on depressive disorders, the number of females was higher than males, to compare two equally-sized groups a casual selection of 17 females was conducted. The following differences were observed: *DEQ factors*: Women showed higher scores than men on dependency (Mann Whitney *U* = 79, adjusted z-score = −2.25, *p* = 0.024); *SWAP-200 factors*: Men showed higher scores than women on the Obsessive PD factor (Mann Whitney *U* = 73, adjusted z-score = −2.46, *p* = 0.014) and on the Narcissistic Q factor (Mann Whitney *U* = 53, adjusted z-score = −3.15, *p* = 0.002), whereas women showed higher scores than men on the Dependent-Victimized Q factor (Mann Whitney *U* = 83, adjusted z-score = −2.11, *p* = 0.034).

## Discussion

Similarly to other studies (Blatt et al., 1976; Hirschfeld, 1999; Blatt, 2004, 2008; Luyten and Blatt, 2007; Morey et al., 2010; Mulder et al., 2010), the main goal of the current investigation is to show that depressive disorders are not independent from personality. In particular, results of this study seem to support the hypothesis that a depressive personality configuration—self-criticism particularly—may represent a vulnerability factor that predisposes to experience depression. More generally, the results of our study seem to stress not only the central role of self-criticism, but also the importance of a Borderline PD factor and Dysphoric: Emotionally dysregulated Q-factor in predicting depressive symptomatology.

In greater detail, similarly to literature on depressive disorders (Shea et al., 1990; Zimmerman et al., 1991; Peselow et al., 1994; Gabbard, 1995; Corruble et al., 1996; Hirschfeld, 1999; Skodol et al., 1999; Farabaugh et al., 2005; Bagby et al., 2008; Agosti et al., 2009; Gorwood et al., 2010), in the current study participants are characterized by a high presence of traits or full-blown Personality Disorders, probably due to the fact that most of them are adults and have experienced at least three depressive episodes. In more detail, with regard to SWAP PD factors, Cluster C traits are prevalent—similarly to findings in literature (Skodol et al., 1999; Fava et al., 2002)—whereas with regard to Q factors, the high presence of the Dysphoric Q factor is of particular interest.

With respect to the correlation between DEQ scales and SWAP factors, both self-criticism and dependency show a positive correlation with the Dependent PD factor. The anaclitic dimension and Dependent Personality Disorder seem to share characteristics such as: The need to be taken care of, the fear of separation, the difficulty in expressing disagreement with others, the tendency to worry about being left to take care of oneself, feelings of loneliness, abandonment, helplessness. Although the positive association between self-criticism and the Dependent PD factor could strike us as contrary to expectations, it might reflect the fact that self-critical individuals may exhibit considerable dependency in the few close relationship that they manage to establish. Similarly, as Zuroff and Mongrain (1987) argued, high levels of introjective depression can be reported by dependent subjects. Moreover, both dependency and self-criticism are positively correlated with the Avoidant PD factor. If the anaclitical dimension could be characterized by hypersensitivity to rejection and criticism, the introjective dimension might be related to the tendency to interpret a wide variety of events as evidence of one's inadequacies, responding to episodes of rejection, failure and loss with self-blame, self-criticism and the avoiding of interpersonal involvement (Zuroff and Mongrain, 1987; Blatt and Homann, 1992; Zuroff and Fitzpatrick, 1995). Similarly to borderline psychopathology (Blatt and Shichman, 1983), it might be hypothesized that the Avoidant PD could likewise include two separate types of avoidant phenomena that may occur in the anaclitic or the introjective configuration, respectively. Finally, contrary to literature that shows a very specific association between schizoid traits and measures of autonomy and self-criticism (Ouimette et al., 1994; Oasi, 2015), in this study the Schizoid PD factor is positively correlated with dependency.

With regard to SWAP Q factors, the positive correlation between self-criticism and the Dysphoric: Emotionally dysregulated Q factor could be explained by the presence, in these participants, of traits that reflect emotions that spiral out of control, struggles with genuine suicidal wishes, an inability to soothe or comfort themselves when distressed, a tendency to feel life has no meaning, a tendency to make repeated suicidal threats or gestures and to “catastrophize,” a tendency to be needy and dependent, and a tendency to engage in self-mutilating behavior (Westen and Shedler, 1999a).

With regard to the association between SWAP factors and depressive measures, firstly, no PD factor is associated to the HAMD—unlike the BDI-II and its subscales—probably due to the kind of items that the HAMD includes, focused on somatic and neurovegetative symptoms of depression. Secondly, the positive correlation between the Dysphoric: Emotionally dysregulated Q factor as well as the negative correlation between both the Obsessional Q factor and the High Functioning Depressive Personality factor with all depressive measures are of particular interest; these Q factors include several psychological strengths that could be affected by the depressive state (i.e., item 2: Is able to use his/her talents, abilities, and energy effectively and productively; 19: Enjoys challenges; takes pleasure in accomplishing things; 68: Has a good sense of humor).

The series of regression analyses shows that, consistently with literature (Blatt et al., 1976; Blatt, 2004), only self-criticism seems to be able to predict both cognitive and somatic dimensions of BDI-II^3^. In addition, the Borderline PD factor as well as the Dysphoric: Emotionally dysregulated Q factor seem to be important in predicting severity of BDI-II. A chronic affective instability or emotional dysregulation, the tendency to experience negative emotions quite intensely—that are typical of both the Borderline PD factor (Tragesser et al., 2007) and the Dysphoric: Emotionally dysregulated Q factor—could enhance the vulnerability to develop depressive symptoms. It is widely known that borderline patients experiment a particular quality of depression (Gunderson and Phillips, 1991; Stanley and Wilson, 2006; Levy et al., 2007; Gunderson et al., 2008; Silk, 2010; Levenson et al., 2012), characterized by earlier onset, chronic, recurrent, and progressive (Skodol et al., 1999), “emptiness, loneliness, diffuse negative affectivity (including anger, loneliness, fear, and desperation), markedly inconstant self-concept and self-esteem, dependency, fears of abandonment, and related interpersonal concerns” (Westen et al., 1992, p. 388). Nevertheless, borderline depression can also be related to self-criticism because of identity disturbances, rather than interpersonal conflicts or affect-regulatory problems (Levy et al., 2007). In this sense, similarly to the results emerged in a study by Ouimette et al. (1994), this data might—considered as a whole—support the possibility of an introjective borderline subtype—as hypothesized by Blatt and Shichman (1983)—that characterizes individuals that exhibit primary conflicts over self-worth and autonomy.

As in Luyten et al. (2007), women show higher scores than men on dependency. This finding seems to be consistent with research that confirms the tendency of women to develop a particular sensitivity regarding issues related to interpersonal concerns (Blatt and Shichman, 1983; Cramer et al., 1988; Blatt and Homann, 1992; Blatt, 2004). Contrary to findings in literature (Silverstein et al., 2013), women don't appear to show a prevalence of somatic depressive symptoms compared to men, and men don't appear to show a higher presence of cognitive depressive symptoms compared to women. Similarly to findings by Carter et al. (1999), in these participants men show higher scores than women on Obsessive PD factor. This result might be considered consistent with the hypothesis that men tend to be more focused on the issues of self-definition, autonomy, self-control, self-worth, and identity, as suggested by Blatt (Blatt and Shichman, 1983). It is interesting to note the higher scores of women than men on the Dysphoric: Dependent Victimized Q factor, that includes individuals who appear to be much more disturbed than those in the axis II dependent category (Westen and Shedler, 1999a). However, results regarding gender differences in the present study should be interpreted with caution owing to the small number of participants in each group.

Our research presents some limitations. The moderate sample size—due to the difficulty in recruiting an appropriate sample of participants in the midst of an unipolar depressive episode^4^—may have limited the validity of the results obtained. Results should be considered as preliminary, exploratory and partial; replication of the present findings in a larger sample is needed. The stability over time of some findings should be verified by longitudinal studies. Furthermore, as suggested by Luyten et al. (2007), prospective studies are needed to investigate the temporal relationship between depression and personality dimensions of dependency and self-criticism. Future studies should also incorporate interviews with significant others, such as family members and professionals (e.g., social workers).

Despite its limitations, data of the present investigation might have important clinical implications for the treatment of depressive disorders. Firstly, although the clinical relevance of these results remains to be further established, these findings are of particular importance in light of the fact that other studies have demonstrated the clinical validity and utility of the depressive personality construct (Phillips et al., 1990; Huprich, 1998, 2012; Phillips and Gunderson, 1999; Westen and Shedler, 1999a,b; Ryder et al., 2006, 2010; Chamberlain and Huprich, 2011; Campos, 2013). Secondly, as in other research (Ouimette et al., 1994; Luyten et al., 2007), this investigation seems to suggest that self-criticism might be involved in a broader range of psychopathology, raising the possibility that this construct may be a relatively non-specific marker of general psychopathology.

## Ethics statement

This study was carried out with written informed consent by all subjects and was approved by the Ethics Committee of the Azienda U.S.L. Rome E.

## Author contributions

All authors helped to conceive and plan the study and prepared and approved the final manuscript. FS conducted the data collection and produced the first draft of the final manuscript. SR conducted the analyses. VL and RZ supervised the data collection. SV set the tables and carefully read the final version of the manuscript. OO formalized the rationale for the study, led on the interpretation of findings, and revised the final version of the manuscript.

### Conflict of interest statement

The authors declare that the research was conducted in the absence of any commercial or financial relationships that could be construed as a potential conflict of interest.

## Footnotes

^1^As stated by Brown et al. (1995), despite their equivalent assessment of severity of depression, the BDI and the HAMD emphasize different dimensions of depression. Schneibel et al. (2012) stress that they should be regarded as two complementary rather than redundant or competing measures: While the HAMD accentuates somatic and behavioral symptoms of depression, the BDI emphasizes the subjective experience of depression; they also suggest using both instruments, particularly for more severely disturbed individuals.

^2^Westen and Shedler (1999b) found that a large number of patients—over 20% of the total sample—were included in the SWAP-200 Dysphoric Q-Factor. Performing a Q-analysis procedure, they were able to identify five subgroups of patients labeled as follows: (1) Dysphoric: avoidant; (2) Dysphoric: High functioning depressive; (3) Dysphoric: Emotionally dysregulated; (4) Dysphoric: Dependent-masochistic; (5) Dysphoric: Hostile-externalizing. Moreover, investigating the convergent validity of the SWAP-200 Dysphoric Q-factor, Diener and Hinselroth (2004) found several significant correlations between the Dysphoric Q-factor and a number of the clinician ratings of depressive symptomatology.

^3^Similarly to Desmet et al. (2006)'s results, this finding raises the question whether self-criticism and BDI-II include items that overlap and doesn't support the hypothesis that dependency is specifically associated with the somatic dimension of BDI-II.

^4^In the recruiting phase we excluded only the bipolar patients within the depressive disorders spectrum. We are aware that this criterion could present possible risks. However, in our opinion the assessment of the unipolar patient—in particular in the midst of a depressive episode—should not present particular problems in terms of reliability. In other words, personality assessment and psychiatric disorders, when not delusional or in the acute phase, are not incompatible (see, for example, Lingiardi and McWilliams, 2015).
